# Signification of Oxygen

**Published:** 1878-02

**Authors:** 


LIQUEFACTION OF OXYGEN.—Students of chemistry will be interested by the following telegram from Professor Pictet, of Geneva, which was received this week by Professor Tyndall: "Oxygène liquifié samedi par acides sulfureux et carboniques combinès. Pression, 320 atmospherès. Temperature, 100 deg. Centigrade de froid." Hitherto, all attempts to liquefy oxygen have failed.—*Brit. Med. Jour.*

